# Epileptic Tissue Localization through Skewness-Based Functional Connectivity in the High-Frequency Band of Intracranial EEG

**DOI:** 10.3390/bioengineering10040461

**Published:** 2023-04-10

**Authors:** Mu Shen, Lin Zhang, Yi Gong, Lei Li, Xianzeng Liu

**Affiliations:** 1 School of Artificial Intelligence, Beijing University of Posts and Telecommunications, Beijing 100876, China; zhanglin@bupt.edu.cn (L.Z.); leili@bupt.edu.cn (L.L.); 2 School of Information and Communication Engineering, Beijing Information Science and Technology University, Beijing 100096, China; gongyi@bistu.edu.cn; 3 Department of Neurology, Peking University International Hospital, and Peking University Clinical Research Institute, Beijing 102206, China; liuxianzeng2004@sina.com

**Keywords:** connectivity, epilepsy, high-frequency activity, high-frequency oscillations, intracranial EEG, localization

## Abstract

Functional connectivity analysis of intracranial electroencephalography (iEEG) plays an important role in understanding the mechanism of epilepsy and seizure dynamics. However, existing connectivity analysis is only suitable for low-frequency bands below 80 Hz. High-frequency oscillations (HFOs) and high-frequency activity (HFA) in the high-frequency band (80–500 Hz) are thought to be specific biomarkers in epileptic tissue localization. However, the transience in duration and variability of occurrence time and amplitudes of these events pose a challenge for conducting effective connectivity analysis. To deal with this problem, we proposed skewness-based functional connectivity (SFC) in the high-frequency band and explored its utility in epileptic tissue localization and surgical outcome evaluation. SFC comprises three main steps. The first step is the quantitative measurement of amplitude distribution asymmetry between HFOs/HFA and baseline activity. The second step is functional network construction on the basis of rank correlation of asymmetry across time. The third step is connectivity strength extraction from the functional network. Experiments were conducted in two separate datasets which consist of iEEG recordings from 59 patients with drug-resistant epilepsy. Significant difference (p<0.001) in connectivity strength was found between epileptic and non-epileptic tissue. Results were quantified via the receiver operating characteristic curve and the area under the curve (AUC). Compared with low-frequency bands, SFC demonstrated superior performance. With respect to pooled and individual epileptic tissue localization for seizure-free patients, AUCs were 0.66 (95% confidence interval (CI): 0.63–0.69) and (0.63 95% CI 0.56–0.71), respectively. For surgical outcome classification, the AUC was 0.75 (95% CI 0.59–0.85). Therefore, SFC can act as a promising assessment tool in characterizing the epileptic network and potentially provide better treatment options for patients with drug-resistant epilepsy.

## 1. Introduction

Epilepsy is characterized by recurrent seizures and affects about 70 million people worldwide [1]. For nearly one-third of refractory patients, seizures cannot be fully controlled by antiepileptic drugs [2]. In order to achieve seizure freedom for those patients, clinical treatment including surgical resection, laser ablation and responsive neurostimulation (RNS) of epileptic tissue are considered during intracranial electroencephalography (iEEG) monitoring [3,4,5]. However, effective treatment depends on accurate localization of epileptic tissue, which is a major challenge in clinical practice.

Epileptic tissue localization can be directly conducted based on invasive iEEG signals [6,7,8,9]. In recent decades, with the advancement of recording technology and understanding of ictogenesis, automatic epileptic tissue localization has become feasible. Emerging evidence supports the notion that epilepsy is a network disease associated with functional brain network alteration among interconnected nodes [4]. Network analyses derived from inter-regional relationships in multiple frequency bands have revealed the differing connectivity strength and synchronizability in patients with different surgical outcomes and RNS effects [2,3,4,10]. Meanwhile, high-frequency oscillations (HFOs) and high-frequency activity (HFA) in the high-frequency band (80–500 Hz) have been demonstrated as promising biomarkers in epilepsy [5,11]. These prominent transient high-frequency events occur in a much smaller and experimentally tractable scale, and their generation is associated with normal and epileptic processes at both the cellular and network levels [11]. Various properties of HFOs/HFA such as rates and morphology have been extensively studied in both epileptic and normal tissue and utilized in epileptic tissue localization and surgical outcome evaluation [6,7,9,12]. Therefore, network analysis in the high-frequency band is expected to provide new insight into epilepsy.

However, existing connectivity analysis is mainly designed for the low-frequency band below 80 Hz. Since iEEG signals in the low-frequency band are continuous over time, connectivity analysis is usually based on amplitude or phase correlations across channels or brain regions [2,3,10,13,14]. However, the same analysis cannot be directly extended to iEEG signals in the high-frequency band owing to two concerns. First, typical HFOs in the high-frequency band only last tens of milliseconds [15]. The majority of recording is baseline activity, which does not bear information of the epileptic network [7]. Second, HFO rates and amplitudes vary significantly across brain structures and functions [12,16]. Such variability poses practical problems in HFO/HFA detection and validation [5]. Despite the existence of several studies focusing on network analysis in the high-frequency band [4,14,17,18], the two concerns mentioned above are not fully resolved and limit their utility in improving epileptic tissue localization and surgical outcome evaluation.

To deal with these problems, we applied skewness-based functional connectivity (SFC) in the high-frequency band. SFC comprises three main steps. In the first step, skewness is employed as a quantitative measurement to characterize the amplitude distribution asymmetry between transient HFOs/HFA and baseline activity. High skewness values are correlated with pathological HFOs, as well as informative background activity, in epileptic tissue [8,9,19,20]. In the second step, a functional network is constructed on the basis of rank correlation to capture the non-linear and long-term connectivity among different channels. To the best of our knowledge, this is the first time that skewness across time has been utilized in network analysis, and previous studied mainly focused on the skewness features for each channel separately. In the third step, we extracted the connectivity strength for each channel within the network for epileptic tissue localization and surgical outcome evaluation.

Therefore, after suppression of the influence of baseline activity and extended correlation across time in the high-frequency band, we hypothesized that SFC is able to provide diagnostic information on the epileptic network. Experiments were conducted on two separate datasets including a total of 59 patients. Epileptic tissue localization and surgical outcome evaluation results were analyzed with respect to the receiver operating characteristic (ROC) curves and the area under the curve (AUC).

## 2. Methods

**Figure 1:**
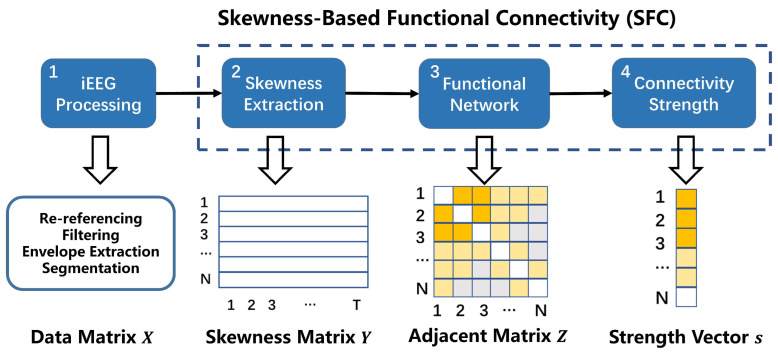
Schematic diagram of the skewness-based functional connectivity analysis (steps 2–4) in the high-frequency band after iEEG signal processing (step 1). In step 2, based on the processed data matrix X, the skewness matrix Y is extracted with size N×T, representing channel number and time, respectively. In step 3, the functional network is constructed based on rank correlation among channels and characterized by the adjacent matrix Y with size N×N. In step 4, by summing up the edge weights between a given channel and the other channels, the strength vector (s) with size N×1 is used for epileptic tissue localization and surgical outcome evaluation.

**Figure 2:**
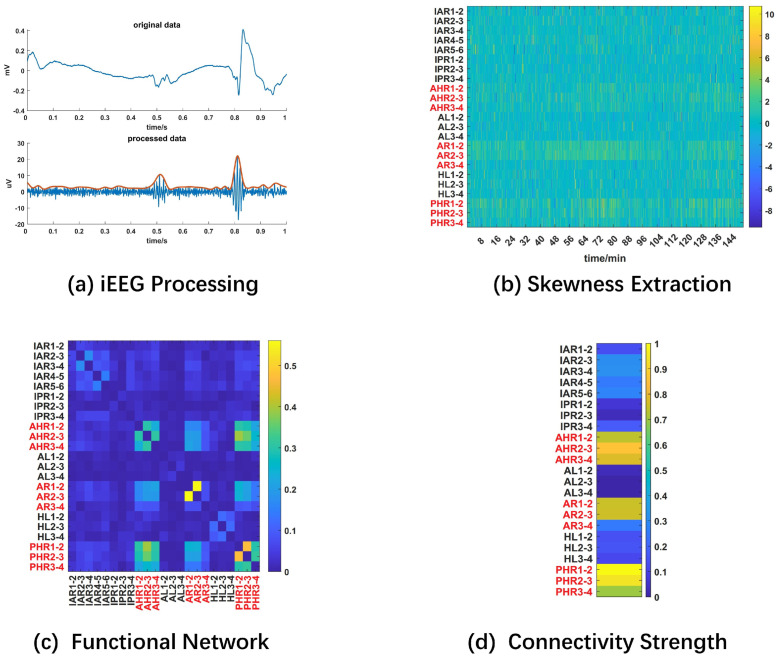
Example patient (P − 1 in dataset 1) in the skewness-based functional connectivity analysis in the high-frequency band. Channels within epileptic tissue are marked in red. (**a**) iEEG signals before and after processing. The upper envelope in red is used for skewness extraction. (**b**) The skewness matrix along channels over time. (**c**) The adjacent matrix that characterizes the epileptic network. (**d**) The connectivity strength vector for epileptic tissue localization and surgical outcome evaluation.

A schematic diagram of SFC analysis is presented in Figure 1. An example of the SFC analysis pipeline for patient P − 1 in dataset 1 is shown in Figure 2.

### 2.1. iEEG Dataset

We retrospectively studied 59 patients with drug-resistant epilepsy from two public datasets [21,22]. Dataset 1 [21] contains 20 patients (14 males and 6 females). The average age is 32.1 ± 11.5 years. A total of 9 patients were diagnosed with temporal lobe epilepsy (TLE), and 11 were diagnosed with extratemporal lobe epilepsy (ETLE). For TLE patients, depth electrodes (1.3 mm in diameter, 8 contacts 1.6 mm in length and 5 mm spacing between contact centers) were implanted stereotactically into the amygdala, the hippocampal head, and the entorhinal and perirhinal cortices bilaterally. Only the 3 most mesial bipolar channels were included for analysis. In ETLE patients, a combination of depth and subdural grid and strip electrodes (contact diameter of 4 mm with a 2.3 mm exposure and 10 mmspacing between contact centers) was placed after craniotomy. Post-implantation magnetic resonance images were used to locate each contact anatomically along the electrode trajectory. The decision for resection surgery was based on non-invasive investigations, as well as on intracranial investigations. For each patient, segments of interictal iEEG recordings taken at least three hours between seizures were extracted during slow-wave sleep with a 2000 Hz sampling rate. All patients underwent surgery, and post-surgical seizure outcome was determined according to the International League Against Epilepsy (ILAE) outcome scale [23]. A total of 13 patients were seizure-free after surgery (ILAE 1). In dataset 2 [22], there are 39 patients in total from multiple centers, of which 20 are males and 17 of which are females; the gender of 2 patients was not recorded. The average age is 34.7 ± 11.1 years (the ages of 3 patients were not recorded). A total of 15 patients were diagnosed with TLE, 6 patients were diagnosed with ETLE and 18 patients were not recorded to have explicit epileptic locations. Dataset 2 contains single or multiple segments of interictal iEEG recordings taken at least from seizures. Signal types include electrocorticography (ECoG) and stereoelectroencephalography (SEEG). The placement of each electrode was determined by the clinical team at each center based on patient history and available non-invasive data. Epileptic tissue was examined based on the comprehensive patient data (non-invasive and invasive) gathered throughout the presurgical evaluation procedure. The range of sampling rates was from 500 Hz to 2000 Hz. To achieve homogeneity of processing parameters across patients in dataset 2, iEEG signals were all resampled with a common sampling rate of 1000 Hz in the FieldTrip toolbox [24]. Treatments included surgical resection, laser ablation and responsive neurostimulation. The outcome was evaluated according to the ILAE and/or the Engel outcome scale [25]. The clinical profiles of patients in the two datasets are listed in Table 1 and Table 2.

### 2.2. iEEG Signal Processing

For patients with multiple segments in dataset 2, only the segment recorded during sleep was selected for analysis, since HFOs occur most frequently and there are fewer muscle artifacts during this period [5]. The iEEG signal processing steps in Figure 1 include:

- Bad channel removal: bad channels marked in the datasets are excluded;
- Rereference: iEEG signals are transformed into a bipolar montage for each electrode to suppress interference caused by severe common-mode noise and outliers during recording;
- Filtering: each iEEG segment is band-passed in the range of 80–500 Hz (dataset 1) or high-passed above 80 Hz (dataset 2) with a finite-impulse-response (FIR) forward–backward filter with stopband attenuation at 60 dB in the Fieldtrip toolbox [24]; furthermore, 60 Hz power line noise and its harmonics are filtered out with two-order Butterworth notch filters with a cutoff frequency of 5 Hz;
- Envelope extraction: the peak upper envelope of iEEG signals is extracted using spline interpolation over local maxima. The upper envelope is used to remove the influence of oscillating components;
- Segmentation: the continuous envelope is further segmented into 1-second epochs without overlap to enhance the temporal resolution.

The raw and processed signals are plotted in Figure 2a. The upper envelope in red was used for further analysis.

### 2.3. Skewness-Based Functional Connectivity Analysis

There are 3 steps in SFC (Figure 1, steps 2–4). Step 2 is skewness extraction. For a time series (x) with n samples, skewness is defined as

(1) $$\mathrm{Skewness}=\frac{\frac{1}{n}\sum_{i=1}^{n}{\left(x_{i}-\bar{x}\right)}^{3}}{(\frac{1}{n}\sum_{i=1}^{n}{\left(x_{i}-\bar{x}\right)}^{2}{)}^{\frac{3}{2}}}$$

where $\bar{x}$ is the mean of x. Instead of direct connectivity quantification based on band-passed signals among channels in the low-frequency band [3,10,13,14,26], skewness is calculated across channels within each epoch to describe the asymmetry in the amplitude distribution between HFOs/HFA and background activity, resulting in a feature matrix (Y) of size N×T, where N is the number of bipolar channels and T is the total recording seconds (Figure 1, step 2, and Figure 2b). High skewness is correlated with pathological high-frequency signals and HFOs [9,19].

In step 3, functional connectivity is calculated based on Spearman rank correlation ($r_{s}$):

(2) $$r_{s}=1-\frac{6\sum_{i=1}^{T}d_{i}^{2}}{T(T^{2}-1)}$$

where $d_{i}$ is the i-th rank difference between all possible pair-row vectors ($y_{i}$) and $y_{j}$ with length T in Y. Then, we obtain the adjacent matrix (Z) of size N×N (Figure 1, step 3, and Figure 2c) to characterize the epileptic network. The diagonal elements in Z are forced to 0. Each channel is considered a node in the network, and the absolute value of Spearman correlation is considered the edge weight between two nodes.

In step 4, the connectivity strength vector (s) is calculated. For each channel, its connectivity strength is defined as the sum of edge weights between itself and the other channels [27], namely the absolute sum for each column elements in Z. Therefore, one patient with N channel recordings is characterized by a connectivity strength vector (s) with a length of N (Figure 1, step 4, and Figure 2d).

### 2.4. Epileptic Tissue Localization

Nodes with connectivity strengths exceeding a certain threshold are labeled ‘epileptic’, whereas nodes with connectivity strengths below the threshold are labeled ‘non-epileptic’. Localization results are categorized as true positive (TP) when the localization result is ‘epileptic’ and the true label is ‘epileptic’; true negative (TN) when the localization result is ‘non-epileptic’ and the true label is ‘non-epileptic’; false positive (FP) when the localization result is ‘epileptic’ and the true label is ‘non-epileptic’; and false negative (FN) when the localization result is ‘non-epileptic’ and the true label is ‘epileptic’. Localization results are quantified at various threshold settings via the receiver operating characteristic (ROC) curve as sensitivity against 1-specificity (false-positive rate, FPR). The area under the curve (AUC) is calculated to evaluate the localization performance. Sensitivity, specificity and FPR are defined as

(3) $$\mathrm{Sensitivity}=\frac{\mathrm{TP}}{\mathrm{TP}+\mathrm{FN}}$$

(4) $$\mathrm{Specificity}=\frac{\mathrm{TN}}{\mathrm{TN}+\mathrm{FP}}$$

(5) $$\begin{array}{cc} \mathrm{FPR}\; & =1-\mathrm{Specificity}\\ \; & =\frac{\mathrm{FP}}{\mathrm{TN}+\mathrm{FP}}. \end{array}$$

In dataset 1, channels within the resected zone (RZ) are labeled for all 20 patients. In dataset 2, channels within the seizure onset zone (SOZ) are labeled in 19 out of 39 patients with RNS or ablation treatment. For these patients, epileptic tissue is defined as the RZ or SOZ, respectively. For the remaining 22 patients in dataset 2 with surgical treatment, both SOZ and RZ are labeled. Since there are usually overlaps between the two regions and post-surgical outcomes are directly determined by surgical resection, the epileptic tissue is defined as the RZ for these 22 patients.

For epileptic tissue localization, both patient-specific and pooled analyses were conducted in this study. In pooled localization, the strength vector derived from each patient is subtracted by mean and normalized between (0,1) to eliminate the influence of channel number and interpatient baseline connectivity strength variability. Then, connectivity strengths from all patients are pooled together for the ROC curve and AUC analysis. In patient-specific localization, the AUC is calculated from the ROC curve of each patient separately. Furthermore, considering that the reliability of epileptic tissue location for each patient is dependent on the surgical outcome, we separated 59 patients into two groups. The first group includes patients with seizure-free outcomes (ILAE class 1–2 or Engel class 1). For these patients, ’true’ epileptic tissue is equal to or contained within the RZ or SOZ. The second group includes patients with poor outcomes (ILAE class 3–6 or Engel class 2–4). For these patients, the SOZ or RZ contains only a part of ’true’ epileptic tissue, since they still suffer from seizures after treatment.

### 2.5. Comparison with the Low-Frequency Band and Direct Extension to the High-Frequency Band

Connectivity analysis was also conducted in the delta (1–4 Hz), theta (4–8 Hz), alpha (8–13 Hz), beta (13–30 Hz) and gamma (30–80 Hz) bands, with direct extension to the high-frequency band. In contrast to SFC, the connectivity matrix is calculated with Spearman correlation directly based on the filtered signals within each epoch and averaged over time [13]. Then, the strengths of nodes are calculated from the adjacent matrix in the same way.

### 2.6. Statistical Analysis

Distributions are represented by box plots with median, interquartile range (IQR), 1.5 times of IQR and whiskers for outliers. One-way ANOVA is used to test the mean difference of node connectivity strengths between epileptic and normal tissue. A two-sided *p*-value of 0.05 is considered the significance level, and 95% confidence intervals (CIs) of AUCs in pooled analysis are estimated via the bootstrapping method with 1000 iterations. The 95% CIs of patient-specific AUCs are estimated via t-statistics. The effect size is quantified with Cohen’s d [28] in terms of paired individual AUCs between SFC and the other frequency bands.

## 3. Results

### 3.1. Connectivity Strengths between Epileptic and Normal Tissue

**Figure 3:**
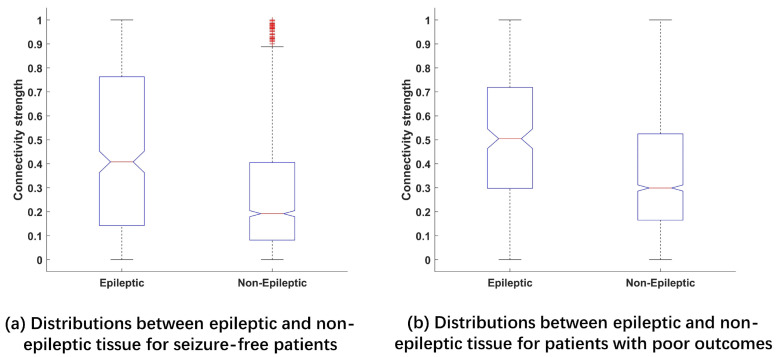
Connectivity strength distributions between epileptic and non-epileptic tissue: (**a**) seizure-free patients; (**b**) patients with poor outcomes. Significant difference (one-way ANOVA, *p* < 0.001) was found in both cases.

There are 468 epileptic vs. 1584 non-epileptic channels for seizure-free patients and 258 epileptic vs. 1950 non-epileptic channels for patients with poor outcomes. In Figure 3, connectivity strengths of epileptic channels are significantly higher (*p* < 0.001) than non-epileptic channels.

### 3.2. Pooled Epileptic Tissue Localization

**Figure 4:**
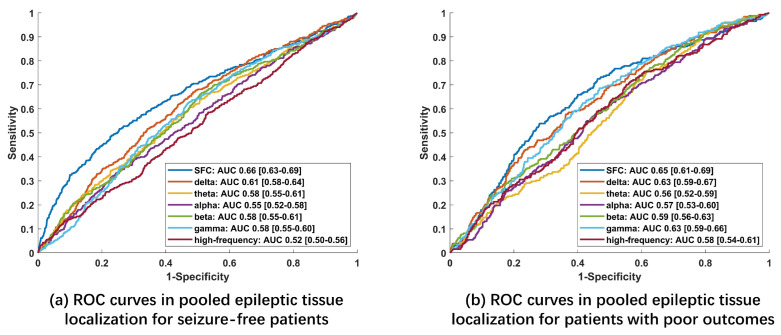
ROC curves and corresponding AUCs with 95% CIs in brackets in pooled localization for (**a**) seizure-free patients and (**b**) patients with poor outcomes.

Next, these connectivity strength differences are directly utilized to localize epileptic channels. The ROC curves and corresponding AUCs are illustrated in Figure 4.

In Figure 4a, the AUCs are 0.66 (95% CI: 0.63–0.69) for SFC, 0.61 (95% CI: 0.58–0.64) for delta, 0.58 (95% CI: 0.55–0.61) for theta, 0.55 (95% CI: 0.52–0.56) for alpha, 0.58 (95% CI: 0.55–0.61) for beta, 0.58 (95% CI: 0.55–0.60) for gamma and 0.52 (95% CI: 0.50–0.56) for the high-frequency band. Under low FPR (<0.4), SFC outperforms the other bands by a large margin. For example, when FPR is 0.2, the sensitivity is above 0.4. With FPR continuously increasing, the sensitivities across different bands and SFC are close to each other. Figure 4a indicates that for seizure-free patients, a proportion of epileptic tissue can be directly identified by SFC, which is characterized by higher connectivity strengths compared with the other part of the epileptic tissue and normal tissue, consistent with the fact that the ’true’ epileptic tissue is within the RZ or SOZ. In contrast, the sensitivities of the other bands are much lower under low FPR, meaning that connectivity strengths in these bands are less sensitive to the epileptic tissue.

In Figure 4b, the AUCs are 0.65 (95% CI: 0.61–0.69) for SFC, 0.63 (95% CI: 0.59–0.67) for delta, 0.56 (95% CI: 0.52–0.59) for theta, 0.57 (95% CI: 0.53–0.60) for alpha, 0.59 (95% CI: 0.56–0.63) for beta, 0.63 (95% CI: 0.59–0.66) for gamma and 0.58 (95% CI: 0.54–0.61) for the high-frequency band. Compared with Figure 4a, under low FPR (<0.2), SFC and the other bands are all characterized by lower sensitivities around chance level, indicating that less high-connectivity strengths are located in the epileptic tissue. When FPR is between 0.2 and 0.6, the sensitivities of the SFC, delta and gamma bands increase quickly compared with the theta, alpha and beta bands. SFC maintains better localization ability than the other bands. The trend during this interval indicates that more medium-connectivity strengths are located within epileptic tissue. A possible explanation is that for patients with poor outcomes, only a part of ’true’ epileptic tissue is resected or treated, whereas the remaining ’true’ epileptic tissue still leads to seizures.

### 3.3. Individual Epileptic Tissue Localization for Seizure-Free Patients

**Figure 5:**
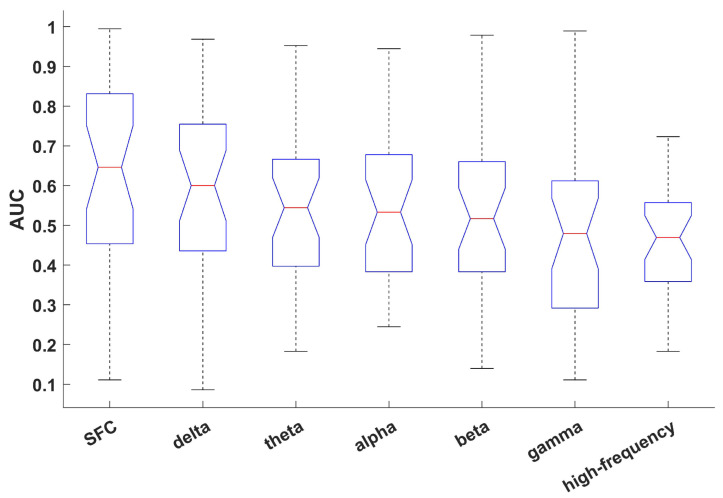
Distributions of individual AUCs for seizure-free patients.

In clinical practice, patient-specific localization is also important because it may provide straightforward diagnostic ability for each patient. Individual localization results for seizure-free patients in terms of AUCs are illustrated in Figure 5 and Table 3.

Compared with Figure 4a, the average AUCs decrease with a wider range of 95% CIs due to the variability caused by different types/numbers of electrodes, recording duration and locations of epileptic tissue across patients. Localization results of SFC rank first in both patient-specific and pooled analysis. The delta band also maintains good performance in both cases. Localization results of the gamma band drop in patient-specific analysis relative to pooled analysis.

In Figure 5 and Table 3, for 32 seizure-free patients, the average individual AUCs are 0.63 (95% CI: 0.56–0.71) for SFC, 0.59 (95% CI: 0.50–0.67) for delta, 0.56 (95% CI: 0.48–0.63) for theta, 0.54 (95% CI: 0.47–0.60) for alpha, 0.52 (95% CI: 0.44–0.60) for beta, 0.47 (95% CI: 0.39–0.55) for gamma and 0.46 (95% CI 0.41–0.51) for the high-frequency band. SFC achieves better localization performance than the other bands. A medium effect size (Cohen’s d > 0.5) was found for SFC against the gamma (0.65) and high-frequency bands (0.79). A small effect size (Cohen’s d > 0.2) was found for SFC against the theta (0.27), alpha (0.31) and beta (0.42) bands. The effect size between SFC and the delta band is 0.17. The mean and lower and upper quartiles of SFC are also higher than for the other bands.

### 3.4. Surgical Outcome Evaluation

**Figure 6:**
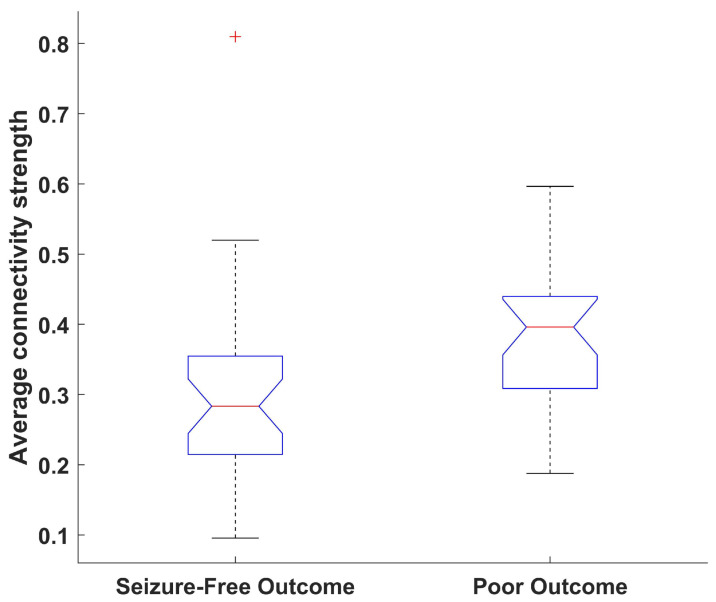
Average connectivity strength distributions outside the epileptic tissue between patients with seizure-free outcomes and patients with poor outcomes. A significant difference (one-way ANOVA, *p* < 0.01 (0.0069)) was found.

**Figure 7:**
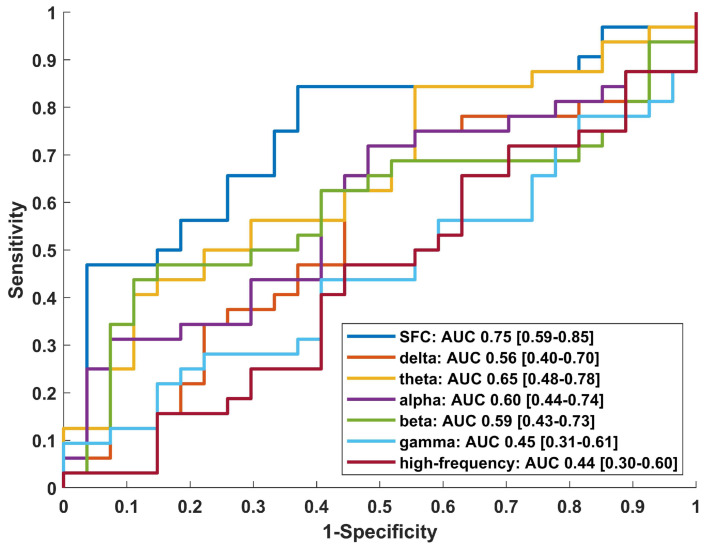
ROC curves and corresponding AUCs with 95% CIs in brackets for surgical outcome classification (seizure-free vs. poor).

Based on the localization characteristics presented in Figure 4a,b, the connectivity strength distribution differences between seizure-free patients and patients with poor outcomes provide predictive information on the surgical outcomes. According to the network hypothesis proposed in [4], complete removal of nodes in the epileptic network within the RZ or SOZ results in seizure freedom, whereas residual nodes in the decentralized epileptic network outside the RZ or SOZ still produce seizures. It can be inferred that the average connectivity strengths outside the epileptic tissue for patients with poor outcomes would be higher than seizure-free patients, since there are still ’true’ epileptic nodes remaining. As illustrated in Figure 6, a significant difference (*p* < 0.01) was found between the average connectivity strength for the two groups of patients. Therefore, similar to pooled analysis, after subtraction by mean and normalization between (0,1), the minus average connectivity strength outside the epileptic tissue for each patient is used to predict two kinds of surgical outcomes (seizure-free vs. poor). The results are illustrated in Figure 7.

In Figure 7, the AUCs are 0.75 (95% CI: 0.59–0.85) for SFC, 0.56 (95% CI: 0.40–0.70) for delta, 0.65 (95% CI: 0.48–0.78) for theta, 0.60 (95% CI: 0.44-0.74) for alpha, 0.59 (95% CI: 0.43–0.73) for beta, 0.45 (95% CI: 0.31–0.61) for gamma and 0.44 (95% CI: 0.30–0.60) for the high-frequency band. When FPR is less than 0.1, the sensitivity of SFC is above 0.4. When FPR is 0.4, the sensitivity of SFC exceeds 0.8. However, due to the limited number of patients (59), the range of 95% CIs is relatively large (≥0.26).

## 4. Discussion

### 4.1. Epileptic Tissue Localization Based on Connectivity in Different Frequency Bands

In recent years, a number of studies have been conducted to reveal the utility of functional connectivity in characterizing seizure dynamics and evaluating surgical outcomes [3,10,13,29]. The frequency ranges used in these studies are generally below 80 Hz [3,10,13] or up to 105 Hz [29]. Compared with oscillations in low-frequency bands, HFOs in the high-frequency band only last tens of milliseconds [15]. Direct extension of commonly used functional connectivity measurement into the high-frequency band is not informative, as illustrated in Figure 4, Figure 5 and Table 3. Two problems are associated with conducting effective connectivity analysis in the high-frequency band reflected by local neural activities need to be addressed: short-transience and the variability of occurrence time and amplitudes of HFOs/HFA.

Some attempts have been made to address these problems [14,26]. In [14], the authors proposed a directed functional network analysis based on a multivariate autoregressive model to quantify HFO propagation between two channels. However, the localization results are sensitive to model order, which varies among bands. The second problem is partly solved by parameter tuning. However, the first problem still exists. In this study, two problems are considered: rather than analysis of band-passed signals in the high-frequency band, skewness is extracted to quantify the intensity of HFOs/HFA compared with background activity within an epoch, which constitutes the basic element for connectivity analysis. Then, connectivity is constructed across time to capture the channel-wise relationships rather than the average. SFC can be interpreted as an indirect way to observe HFOs/HFA in the high-frequency band on a large time scale.

With respect to localization results in pooled analysis, the delta and gamma bands perform better than the theta, alpha and beta bands. These results are consistent with previous findings [14,30,31,32]. Widespread increased delta activity during sleep is correlated with plastic changes, interictal spikes and seizure focus [31]. Gamma oscillations are associated with SOZ and the pathological network [14,32]. However, for individual localization results, the average AUC of the gamma band drops below the chance level (0.5). It is possible that variability in the channel number and location of epileptic tissue limit its localization ability. In contrast, the average AUCs of SFC and delta band maintain better localization performance than the other bands. Since the delta wave is dominant in amplitude and duration during sleep, the connectivity analysis is more robust than for the other bands. However, it lacks the ability to predict post-surgical outcomes, probably because it functions in a large brain area and is not specific enough to discriminate the difference outside the epileptic tissue. For SFC, it achieves the highest AUCs across pooled and individual localizations, as well as surgical outcome classification. Direct comparison with the gamma and high-frequency band supports the notion that SFC can effectively overcome the two problems in connectivity analysis of the high-frequency band.

In terms of effect size in individual localization for seizure-free patients, SFC shows advantages over the theta, alpha, beta, gamma and high-frequency bands (Cohen’s d ≥ 0.2). The effect size compared with the delta band is smaller (Cohen’s d is 0.17). It is possible that the delta and high-frequency bands reflect different aspects of the epileptic network and potentially provide complementary information.

### 4.2. Comparison with Network Analysis Based on HFOs

Transient HFOs can be detected via various detection algorithms [5]. An epileptic network can also be constructed purely based on discrete HFOs, regardless of the background and baseline activity [4,17]. The authors of [17] constructed HFO networks by measuring statistically significant HFO delays among the channels and found that resecting source channels is not superior to resecting channels with the highest rates of HFOs. In [4], two kinds of FR networks (rate-distance and mutual information networks) were constructed. The rate distance network was constructed based on multiplication by the FR rate and the Euclidean distance between contacts. The MI network was based on the timing of FR trains. Results supported the epileptic network hypothesis of ictogenesis. Two problems in high-frequency band connectivity analysis were also solved. The first problem was solved by HFO detection, which explicitly separates HFOs from the other activity. The second problem was tackled by information extraction from timing, rates and mutual information of detected HFOs.

There are some fundamental differences between HFO network analysis and SFC analysis. The advantage of HFO network analysis is that it is based on pure HFOs after rejecting falsely detected HFOs, including sharp spikes and artefacts. However, HFO detection itself is challenging. First, variability in detection criteria and the visual review process could lead to different detection results and result in inevitable analysis bias. There are various definitions of HFOs in automatic detection algorithms [5]. Visual review is usually necessary to further validate detected HFOs [4,17]. Second, the recommended sampling rate (above 2000 Hz) for recording of HFOs in the 80–500 Hz range [15] sometimes cannot be met. For example, the sampling rates for 32 out of 39 patients in dataset 2 are below 2000 Hz, causing unreliable, biased or inapplicable results for HFO network analysis. In contrast, SFC analysis is based on the amplitude distribution asymmetry in the entire high-frequency range, which incorporates pure and falsely detected HFOs, as well as background activity. The requirement for the sampling rate of SFC is more relaxed, and it can be applied in all patients in dataset 2 by high-pass filtering above 80 Hz or band-pass filtering in the range of 80–500 Hz. Therefore, SFC can be easily applied to other datasets without time-consuming detection and visual examination.

The relationships among background activity, HFA and HFOs are still the subject of active investigation [7,8,9,33]. HFOs on a flat background are more strongly linked to the SOZ than HFOs on an oscillatory background [7]. High-frequency background activity provides distinct and complementary information from detected HFOs [8]. Skewness can be used to identify epileptic channels, and it may not be necessary to separate pure HFOs from false oscillations produced by the filter effect of sharp spikes [9,33]. Aligned with recent advances in HFO network analysis, SFC in the high-frequency band demonstrates its utility in epileptic tissue localization.

### 4.3. Limitation and Future Perspective

Due to the lack of anatomic and functional neuroimaging information, as well as a lack of clinical details, further investigation beyond epileptic tissue localization is not supported. Two cases are especially worth noting. For patients with seizure-free outcomes but low AUCs from SFC, SFC is not informative in epileptic tissue. For patients with poor surgical outcomes, localization results need to be checked carefully, given the information from SFC, to provide potential treatment options. Another limitation is that statistical analysis was not carried out with respect to specific clinical profiles such as the anatomical location of epileptic tissue, age or gender, which are worth consideration with sufficiently large available patient samples in future studies.

Motivated by recent advances supporting the epileptic network hypothesis against the epileptogenic zone hypothesis, this study further confirms the utility of network analysis based on the biomarker in the high-frequency band. In clinical practice, single-channel biomarkers derived from HFOs/HFA, together with network characteristics based on HFOs or SFC, can be combined to provide useful information for presurgical evaluation and reliable and efficient diagnosis.

## 5. Conclusions

SFC aims to characterize functional connectivity in the high-frequency band by skewness extraction. It shows advantages over connectivity in low-frequency bands in epileptic tissue localization and surgical outcome evaluation. Complementary to HFO network analysis, we hope this method can be incorporated in clinical practice to contribute to better treatment options for patients.

## Figures and Tables

**Table 1:** Clinical profiles of patients in dataset 1.

Patient ID	Gender	Age	iEEG Type	Sample Rate	Engel Class	ILAE Class	Treatment	Location of Epileptic Tissue
P-1	M	25	depth, strip	2000	N/A	1	resection	TLE
P-2	M	33	depth	2000	N/A	1	resection	TLE
P-3	F	20	depth	2000	N/A	1	resection	TLE
P-4	F	20	depth	2000	N/A	1	resection	TLE
P-5	M	40	depth	2000	N/A	1	resection	TLE
P-6	M	48	depth	2000	N/A	1	resection	TLE
P-7	M	25	depth	2000	N/A	3	resection	TLE
P-8	F	21	depth	2000	N/A	3	resection	TLE
P-9	M	52	depth	2000	N/A	5	resection	TLE
P-10	M	37	strip, grid	2000	N/A	1	resection	ETLE
P-11	M	36	depth, grid	2000	N/A	1	resection	ETLE
P-12	M	49	depth, grid	2000	N/A	1	resection	ETLE
P-13	M	17	depth, grid	2000	N/A	1	resection	ETLE
P-14	F	46	depth, grid, strip	2000	N/A	1	resection	ETLE
P-15	F	31	strip, grid	2000	N/A	1	resection	ETLE
P-16	F	17	depth, grid	2000	N/A	1	resection	ETLE
P-17	M	30	strip, grid	2000	N/A	5	resection	ETLE
P-18	M	40	depth, strip	2000	N/A	5	resection	ETLE
P-19	M	38	grid	2000	N/A	6	resection	ETLE
P-20	M	17	grid	2000	N/A	5	resection	ETLE

Abbreviations: N/A, not applicable; TLE, temporal lobe epilepsy; ETLE, extratemporal lobe epilepsy.

**Table 2:** Clinical profiles of patients in dataset 2.

Patient ID	Gender	Age	iEEG Type	Sample Rate	Engel Class	ILAE Class	Treatment	Location of Epileptic Tissue
NIH1	F	57	SEEG	1000	1	1	resection	anterior temporal lobe
NIH2	M	31	SEEG	1000	1	1	resection	right post hippocampus, temporal pole/anterior insula
NIH3	F	36	SEEG	1000	1	1	resection	left temporal pole/mesial temporal
NIH4	M	39	SEEG	1000	1	1	resection	right parietal
NIH5	M	41	SEEG	1000	1	1	resection	right frontal
NIH6	F	20	SEEG	1000	3	3	resection	left mesial temporal/amygdala
NIH7	M	46	SEEG	1000	3	4	resection	bitemporal or orbitofrontal
NIH8	M	37	SEEG	1000	2	4	resection	post hippocampus
NIH9	F	16	SEEG	1000	3	4	resection	left frontal, parietal operculum, insula
NIH10	M	25	SEEG	1000	2	3	resection	left insula
NIH11	M	27	SEEG	1000	2	3	resection	left perirolandic
PY18N002	M	62	SEEG	1000	2	2	resection	N/A
PY18N007	F	32	SEEG	1000	4	5	MRgLiTT	N/A
PY18N013	F	24	SEEG	1000	1	1	resection	N/A
PY18N015	F	N/A	SEEG	1000	1	1	resection	N/A
PY19N012	M	48	SEEG	1000	2	3	ablation	N/A
PY19N015	F	23	SEEG	1000	3	4	RNS	N/A
PY19N023	M	32	SEEG	1000	1	1	resection	N/A
PY19N026	F	35	SEEG	1000	1	1	ablation	N/A
jh103	N/A	N/A	ECoG	1000	4	6	resection	right anterior temporal lobe
jh105	N/A	N/A	ECoG	1000	1	1	resection	right temporal lobe
pt1	F	30	ECoG	1000	1	2	resection	right anterior temporal Lobe
pt2	F	28	ECoG	1000	1	1	resection	left anterior temporal lobe
pt3	M	45	ECoG	1000	1	1	resection	right frontal lobe
rns002	F	36	SEEG	2000	3	N/A	RNS	N/A
rns003	M	21	SEEG	2000	3	N/A	RNS	N/A
rns004	M	52	SEEG	500	4	N/A	RNS	N/A
rns005	M	23	SEEG	2000	3	N/A	RNS	N/A
rns006	M	49	SEEG	500	1	N/A	RNS	N/A
rns009	M	48	SEEG	1024	3	N/A	RNS	N/A
rns011	F	24	SEEG	2000	4	N/A	RNS	N/A
rns013	M	25	SEEG	2000	2	N/A	RNS	N/A
rns014	M	36	SEEG	2000	4	N/A	RNS	N/A
rns015	M	27	SEEG	2000	2	N/A	RNS	N/A
umf001	F	37	ECoG	1000	1	1	resection	right anterior temporal lobe
umf002	F	39	ECoG	1000	2	1	resection	right anterior temporal lobe
umf003	M	43	ECoG	1000	3	4	resection	left temporal lobe
umf004	F	23	SEEG	1000	1	1	resection	left anterior medial temporal lobe
umf005	F	32	ECoG	1000	1	1	resection	right anterior temporal lobe

Abbreviations: MRgLiTT, magnetic resonance-guided laser interstitial thermal therapy; RNS, responsive neurostimulation; N/A, not applicable.

**Table 3:** Individual epileptic tissue localization results for seizure-free patients with average AUCs, 95% CIs in brackets and Cohen’s d.

	SFC	Delta	Theta	Alpha	Beta	Gamma	High-Frequency
AUC 95% CI	0.63 [0.56–0.71]	0.59 [0.50–0.67]	0.56 [0.48–0.63]	0.54 [0.47–0.62]	0.52 [0.44–0.60]	0.47 [0.39–0.55]	0.46 [0.41–0.51]
Cohen’s d	-	0.17	0.27	0.31	0.42	0.65	0.79

## Data Availability

The data used in this study were obtained from two public datasets: (1) long-term invasive iEEG recordings from 20 patients, accessible on 23 October 2017 at: https://crcns.org/data-sets/methods/ieeg-1/about-ieeg-1; (2) epilepsy iEEG interictal multicenter dataset, accessible on 9 November 2021 at: https://openneuro.org/datasets/ds003876/versions/1.0.2.

## Abbreviations

The following abbreviations are used in this manuscript:

iEEG	Intracranial electroencephalography
HFOs	High-frequency oscillations
HFA	High-frequency activity
SFC	Skewness-based functional connectivity
SOZ	Seizure onset zone
RZ	Resected zone
AUC	Area under the curve
CI	Confidence interval
FRs	Fast ripples
TLE	Temporal lobe epilepsy
ETLE	Extratemporal lobe epilepsy
ILAE	International League Against Epilepsy
ECoG	Electrocorticography
SEEG	Stereoelectroencephalography
ROC	Receiver operating characteristic
FPR	False-positive rate
IQR	Interquartile range
